# Tension Hydrocele—How Point-of-care Ultrasound Helps in the Emergency Department: A Case Report

**DOI:** 10.5811/cpcem.61719

**Published:** 2026-07-20

**Authors:** Sophia Potalivo, Sophia Fornbacher, Joey Abadilla, Ron Goubert, Eva Tovar Hirashima

**Affiliations:** * University of California Riverside School of Medicine, Riverside, California; † Riverside Community Hospital, HCA Healthcare, Department of Emergency Medicine, Riverside, California

## Abstract

**Introduction:**

Tension hydrocele is a rare but serious complication that can threaten testicular viability. This case report describes how testicular point-of-care ultrasound (POCUS) enabled timely recognition of a large hydrocele compromising testicular perfusion and guided management.

**Case Report:**

A 57-year-old male presented to the emergency department (ED) with painful right scrotal swelling. Testicular POCUS demonstrated a large hydrocele with reduced intratesticular blood flow. A scrotal centesis performed by the emergency physician led to symptom resolution and restoration of normal flow.

**Conclusion:**

Testicular POCUS can rapidly identify impaired perfusion and guide scrotal centesis in the ED, a temporary yet potentially testis-saving intervention.

## INTRODUCTION

Acute scrotal pain is defined as the presence of new-onset moderate or severe pain, swelling, and/or tenderness of the intrascrotal contents.^1^ Male genitourinary complaints, such as acute scrotal pain, account for approximately 0.5–2.5% of all emergency department (ED) visits. These presentations pose a diagnostic challenge because they require rapid differentiation between benign or emergent conditions, with management ranging from reassurance to urgent surgical intervention.^1^ Testicular point-of-care ultrasound (POCUS) offers a rapid, noninvasive means to evaluate these possibly acute scrotal pathologies, distinguishing between vascular and inflammatory etiologies. These include testicular torsion, hydrocele, orchitis, and epididymitis, with reported sensitivities of 94–95%.^2^,^3^ This underscores the essential role of testicular POCUS in acute scrotal evaluation.

Patients with acute scrotal pain may also present with swelling. One differential for swelling is a hydrocele that results from serous fluid accumulation within the tunica vaginalis and may be congenital or acquired. Acquired hydroceles occur in approximately 0.1% of adult men and represent one of the most common causes of scrotal swelling.^4^,^5^ Although traditionally described in the urology literature, tension hydrocele, also termed testicular compartment syndrome, represents a rare cause of impaired testicular perfusion due to excessive hydrocele volume. 6–9 Reported management strategies include bedside scrotal centesis, which was first described in 2008 by Douglas et al.^10^ This case expands the differential diagnosis of atraumatic, acute scrotal pain to include tension hydrocele and highlights the diagnostic and therapeutic value of POCUS in the emergency setting. To our knowledge, this case represents the first report of POCUS-guided scrotal aspiration for this condition, demonstrating a temporizing organ-sparing intervention when urologic consultation is delayed or unavailable.

## CASE REPORT

A 57-year-old man with a history of amphetamine use and known bilateral hydroceles presented to the ED with lower abdominal pain and progressive atraumatic right scrotal swelling over 24 hours. The pain was constant, pressure-like, and rated 10 of 10 in intensity. He denied dysuria, hematuria, or fever.

On examination, the abdomen was flat and nontender. The right hemiscrotum was enlarged, tense, and exquisitely tender, with the testicle nonpalpable. Vital signs were as follows: blood pressure, 129/86 millimeters of mercury; heart rate, 75 beats per minute; respiratory rate, 18 breaths per minute; temperature, 36.7 °C; and oxygen saturation, 95% on room air. Laboratory evaluation, including complete blood count and basic metabolic panel, was unremarkable. Urinalysis was not obtained. Given initial concern for an incarcerated inguinal hernia, a contrast-enhanced computed tomography of the abdomen and pelvis was obtained initially. The study was negative for an inguinal hernia but demonstrated bilateral hydroceles, right greater than left. The right hydrocele measured 6.4 cm and extended into the right inguinal canal.

**Image 1:**
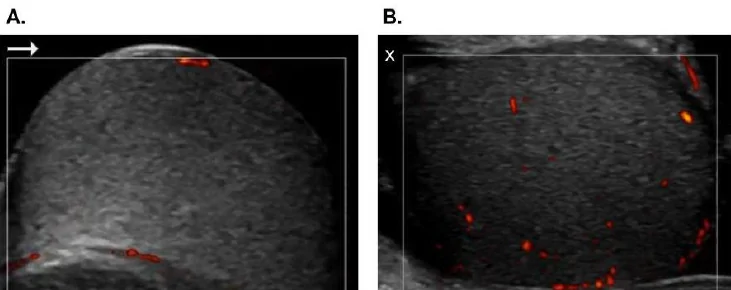
A. Right testicle demonstrates a large hydrocele (arrow) with reduced intratesticular flow. B. Left testicle showing a small hydrocele (x) and preserved intratesticular flow present for comparison. Please note, the thin-lined boxes in these images represent region of applied power Doppler on the ultrasound machine to assess flow.

Testicular POCUS demonstrated a large, simple right hydrocele without internal septations and markedly decreased intratesticular blood flow on power Doppler (Image 1A) when compared to the left (Image 1B). These findings raised concern for a right-sided tension hydrocele. Urology was consulted but unavailable in-house; they agreed with emergent aspiration and recommended outpatient follow-up. Given persistent pain and reduced right testicular perfusion, the emergency physician performed bedside aspiration under ultrasound guidance (Video).

For the scrotal centesis, a linear probe was used under sterile conditions to identify the largest fluid pocket, and with dynamic ultrasound guidance a 20-gauge angiocatheter was inserted into the right hemiscrotum. Approximately 170 mL of clear, straw-colored fluid was aspirated resulting in immediate symptom relief. Samples were sent for cell count, Gram stain, and culture, all of which were unremarkable.

*CPC-EM Capsule*

What do we already know about this clinical entity?

*Hydrocele usually presents as painless scrotal swelling, but it rarely can progress to tension hydrocele, leading to testicular hypoperfusion.*

What makes this presentation of disease reportable?

*Point-of-care ultrasound (POCUS) identified a large hydrocele causing reduced testicular blood flow, which was treated with ultrasound-guided scrotal centesis.*

What is the major learning point?

*Testicular POCUS can rapidly assess blood flow and guide scrotal centesis to relieve pressure.*

How might this improve emergency medicine practice?

*This case highlights the role of testicular POCUS and ultrasound-guided decompression when urologic consultation is delayed.*

**Image 2:**
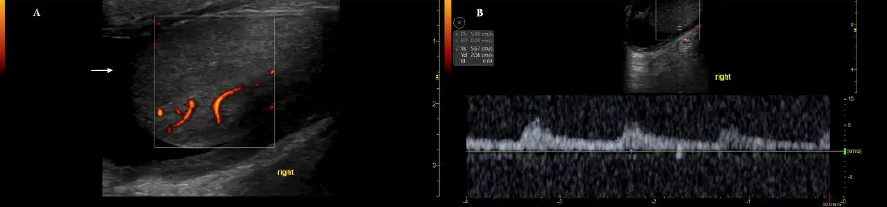
A) Repeat testicular POCUS of the right testicle after scrotal centesis demonstrating markedly reduced hydrocele (longer arrow) and improved intra-testicular blood flow. B) Spectral Doppler demonstrating normalization of perfusion, with a normal, low-resistance waveform. *POCUS*, point-of-care ultrasound.

Repeat testicular POCUS demonstrated restoration of right testicular perfusion, evidenced by improved intratesticular blood flow on power Doppler (Image 2A) and the return of a low-resistance arterial waveform on spectral Doppler (Image 2B).

The patient was discharged with outpatient urology follow-up and return precautions for recurrent pain or swelling. He presented the following day with mild right testicular discomfort. Consultative ultrasound revealed normal bilateral blood flow with right-sided epididymitis. He was treated with ciprofloxacin and continued outpatient follow-up.

## DISCUSSION

Testicular POCUS has transformed the evaluation of acute scrotal conditions, enabling emergency physicians to rapidly assess testicular perfusion and identify surgically significant pathology.^2^,^3^ When evaluating patients with hydroceles and acute scrotal pain, the differential diagnosis should include tension hydrocele, a rare but reversible cause of testicular hypoperfusion.^7^ Unlike testicular torsion, which typically requires emergent surgical intervention, tension hydrocele may respond to simple decompression, resulting in both symptom relief and restoration of testicular perfusion, as demonstrated in this case.

Although no pathognomonic sonographic findings define tension hydrocele, the combination of acute scrotal pain, a large hydrocele, and diminished intratesticular flow on color and/or power Doppler should heighten clinical suspicion.^6^,^8^ In such cases, immediate aspiration is recommended to relieve pressure and restore testicular blood flow, preventing irreversible ischemic injury.^6^,^8^ Testicular POCUS further provides real-time procedural guidance, enabling both diagnostic confirmation and therapeutic assessment through visualization of restored perfusion following decompression.

Definitive management can be scheduled electively, typically within days to weeks, unless there is rapid reaccumulation or persistent pain occurs.^11^,^12^ Hydrocelectomy or aspiration with sclerotherapy are the primary treatment options.^12^ Hydrocelectomy remains the gold standard, with the lowest recurrence rates and is preferred for healthy patients, large or recurrent hydroceles, or when durable resolution is desired. Sclerotherapy is best reserved for high-risk or non-surgical candidates.^12^

This topic is increasingly relevant given the projected national shortage of urologists and the declining availability of on-call urology services in U.S. hospitals, particularly in non-metropolitan and community EDs. Workforce analyses predict a continued decline in per-capita urologist availability through 2037, with fewer than 40% of U.S. counties currently hosting an active urologist.^13^–^15^ National surveys have also identified urology as one of the most frequently unavailable specialties for around-the-clock consultation.^13^ In this context, emergency physicians must increasingly assume an expanded role in the acute management of urologic emergencies. The integration of testicular POCUS and procedural competency, such as bedside decompression of tension hydrocele, reflects a resource-responsive approach to emergency care that mitigates delays and preserves organ function when specialist support is limited.

## CONCLUSION

This case illustrates that tension hydrocele, though uncommon, is a reversible cause of acute scrotal pain and hypoperfusion that can be promptly recognized and treated in the ED. Testicular POCUS serves as both a diagnostic tool for identifying compromised testicular blood flow and a guidance method for therapeutic intervention. Emergency department-performed scrotal centesis provides an effective temporizing measure that can restore testicular perfusion and preserve organ viability while arranging definitive urological care.

## Supplementary Information

Video Procedural video demonstrating ultrasound-guided scrotal centesis for the treatment of tension hydrocele
